# An Evaluation of the Boys Do Cry Suicide Prevention Media Campaign on Twitter: Mixed Methods Approach

**DOI:** 10.2196/49325

**Published:** 2023-09-07

**Authors:** Simone Scotti Requena, Jane Pirkis, Dianne Currier, Mike Conway, Simon Lee, Jackie Turnure, Jennifer Cummins, Angela Nicholas

**Affiliations:** 1 Centre for Mental Health Melbourne School of Population and Global Health The University of Melbourne Melbourne Australia; 2 Centre for Digital Transformation of Health School of Computing and Information Systems The University of Melbourne Melbourne Australia; 3 The Hallway Sydney Australia; 4 Heiress Films Sydney Australia

**Keywords:** help-seeking, masculinity, media campaign, men, men’s health, mental health, self-reliance, social media, suicide prevention, suicide

## Abstract

**Background:**

In most countries, men are more likely to die by suicide than women. Adherence to dominant masculine norms, such as being self-reliant, is linked to suicide in men in Western cultures. We created a suicide prevention media campaign, “Boys Do Cry,” designed to challenge the “self-reliance” norm and encourage help-seeking in men. A music video was at the core of the campaign, which was an adapted version of the “Boys Don’t Cry” song from “The Cure.” There is evidence that suicide prevention media campaigns can encourage help-seeking for mental health difficulties.

**Objective:**

We aimed to explore the reach, engagement, and themes of discussion prompted by the Boys Do Cry campaign on Twitter.

**Methods:**

We used Twitter analytics data to investigate the reach and engagement of the Boys Do Cry campaign, including analyzing the characteristics of tweets posted by the campaign’s hosts. Throughout the campaign and immediately after, we also used Twitter data derived from the Twitter Application Programming Interface to analyze the tweeting patterns of users related to the campaign. In addition, we qualitatively analyzed the content of Boys Do Cry–related tweets during the campaign period.

**Results:**

During the campaign, Twitter users saw the tweets posted by the hosts of the campaign a total of 140,650 times and engaged with its content a total of 4477 times. The 10 highest-performing tweets by the campaign hosts involved either a video or an image. Among the 10 highest-performing tweets, the first was one that included the campaign’s core video; the second was a screenshot of the tweet posted by Robert Smith, the lead singer of The Cure, sharing the Boys Do Cry campaign’s video and tagging the campaign’s hosts. In addition, the pattern of Twitter activity for the campaign-related tweets was considerably higher during the campaign than in the immediate postcampaign period, with half of the activity occurring during the first week of the campaign when Robert Smith promoted the campaign. Some of the key topics of discussions prompted by the Boys Do Cry campaign on Twitter involved users supporting the campaign; referencing the original song, band, or lead singer; reiterating the campaign’s messages; and having emotional responses to the campaign.

**Conclusions:**

This study demonstrates that a brief media campaign such as Boys Do Cry can achieve good reach and engagement and can prompt discussions on Twitter about masculinity and suicide. Such discussions may lead to greater awareness about the importance of seeking help and providing support to those with mental health difficulties. However, this study suggests that longer, more intensive campaigns may be needed in order to amplify and sustain these results.

## Introduction

Suicide is a significant public health issue worldwide, with more than 700,000 people dying by suicide every year [1]. In 2019, the global age-standardized suicide death rate was 2 times greater for males than females [1]. In Australia, males were 3 times more likely to die by suicide than females (18.6 vs 5.8 suicide deaths per 100,000 people) in 2020 [2]. There are multiple factors contributing to a higher suicide risk among men compared to women. These factors include men’s tendency to employ more lethal suicide methods [3], engage in higher levels of problematic alcohol and drug use [4,5], express depression symptoms less frequently [6,7], experience lower social connectedness [8], and seek emotional support less often [9,10] than women. It is noteworthy that symptoms of depression in men are accompanied by social withdrawal and increased isolation [11].

It is also possible that traditional masculinity, the cultural set of norms dictating how men should think and behave [12-14], may underline all these factors that make men more vulnerable to suicide. Cleary [13] suggests that the Western view of masculinity imposes an ideal for men fostering strength and stoicism over the expression of “soft” emotions (eg, sadness). Pirkis and colleagues found that self-reliance, a dominant masculine norm in Western countries, was associated with suicidal thinking in a large sample (n=13,884) of Australian men [15]. Similarly, a large evidence synthesis of 78 studies comprising 19,453 individuals found that adherence to certain masculine norms, such as self-reliance, emotional control, power over women, and being a playboy, was strongly associated with risk factors for suicidal thoughts and behaviors [16]. For this reason, it is crucial to create opportunities for discussions and awareness-building around masculinity and suicide. Media campaigns can contribute by promoting suicide and masculinity-related discussions, such as through social media [17-19]. A systematic review of 20 studies found that suicide prevention media campaigns can improve attitudes toward suicide, including by improving help-seeking; the authors also highlighted the lack of evidence to determine what types of campaign messaging work best in such interventions [20]. A randomized controlled trial of an Australian suicide prevention media campaign focused on challenging adherence to traditional masculine norms found that the campaign influenced conversations about masculinity and suicide on Facebook and Twitter [17,18,21]. However, relatively few other suicide prevention media campaigns, including their follow-on discussions, have been evaluated, particularly ones targeting men [20,22].

In collaboration with an independent film production company (Heiress Films) and an advertising company (The Hallway), and with guidance from several Australian suicide prevention and men’s health community organizations, we developed Boys Do Cry, a suicide prevention media campaign designed to challenge adherence to masculine norms such as self-reliance and encourage help-seeking. At the core of the campaign was a video of a diverse group of Australian men singing an adapted version of the song “Boys Don’t Cry” from the British rock band “The Cure.” The adapted lyrics relate to the importance of men talking to others and reaching out for help if they are struggling emotionally (for the adapted lyrics, see Boys Do Cry [23]). The video also included a well-known Australian First Nations artist and rapper, Dallas Woods, singing part of the lyrics in rap.

The Boys Do Cry campaign was publicly released on November 21, 2021, and the video was made available through Twitter, Facebook, Instagram, TikTok, and YouTube. In addition, a website was developed [24] which served as a hub of resources for the campaign. The website’s home page included a link to the video and pages pointing to practical information about how to reach out for help when struggling emotionally (Get Talking), how to help someone going through a difficult period (Get Listening), and how to get involved in the campaign (Get Cracking). The website also included several links to external support services provided by national nongovernment organizations. Some of these specialized in men’s mental health (eg, MensLine, Mates4Mates, and Brother to Brother), and others were more general (eg, Lifeline, Suicide Call Back Service, and Beyond Blue). The campaign ran for 19 weeks in 2 phases. Phase 1 ran from November 21 to December 31, 2021, and encouraged people to watch the video. Phase 2 ran from January 1 to March 31, 2022, and encouraged people to take action by getting help or talking to a friend who is struggling.

The evaluation of the Boys Do Cry campaign included a randomized controlled trial of the effects of the video on help-seeking intentions that was conducted before the campaign was launched [25]. This study reports on an additional component of the evaluation that aimed to explore the reach, engagement, and discussion themes prompted by the Boys Do Cry campaign on Twitter.

We addressed the study’s aim through 4 research questions:

1. What was the reach of the Boys Do Cry campaign, and how did the Twitter users engage with it?
2. What were the highest- and lowest-performing tweets, and how did the Twitter users engage with them?
3. What was the pattern of Twitter activity for campaign-related tweets over the course of the campaign and in the postcampaign period?
4. What were the key themes of discussion on Twitter prompted by the Boys Do Cry campaign?

## Methods

### Data Collection

**Figure 1:**
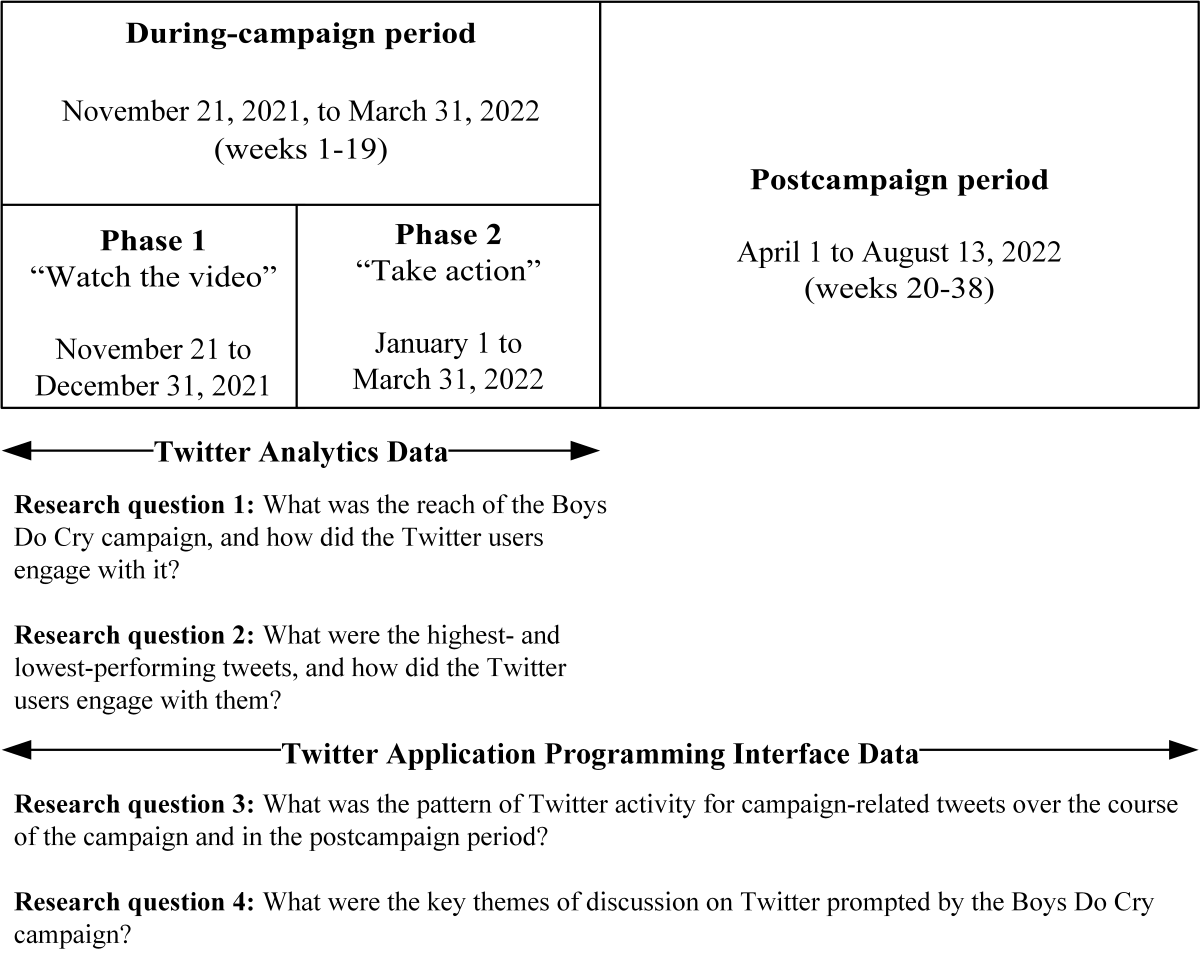
Periods of the 19-week Boys Do Cry suicide prevention campaign aimed at promoting help-seeking among Australian men experiencing mental health difficulties (during-campaign period) versus the 19-week period after the campaign (postcampaign) and the research questions covered by each data source.

To address our research questions, we used Twitter data from 2 sources: (1) Twitter analytics; and (2) Twitter application programming interface (API). Figure 1 shows the time frames for the campaign periods and the research questions covered by each data source.

### Twitter Analytics Data

To address research questions 1 and 2, we downloaded Twitter data using the Twitter analytics tool. This tool is available to all Twitter users; it tracks user metrics such as top performing tweets and mentions, the number of times a tweet is seen on Twitter, and the number of engagements (total number of times users engaged with a tweet). The Twitter analytics tool allows users to freely download their data.

On November 21, 2022, we downloaded monthly tweet activity metrics from the campaign’s hosts (@BoysDoCry2021) for the active campaign period (during campaign), which included the 2 phases of the campaign. We then combined the monthly data files into 1 data set. These data allowed us to identify and describe the campaign’s reach and users’ engagement with the campaign. We described reach as the number of times users saw a tweet posted by @BoysDoCry2021 and engagement as the number of times users interacted with @BoysDoCry2021 tweets (ie, an aggregated measure for clicks anywhere on @BoysDoCry2021 tweets, including retweets, replies, follows, likes, links, hashtags, embedded media, username, profile photo, or tweet expansion) [26]. A total of 247 tweets were posted by the @BoysDoCry2021 account during the campaign. Less than one-quarter of the tweets (51 of 247) were @BoysDoCry2021 replies to other users.

### Twitter Application Programming Interface Data

To address research questions 3 and 4, we used the Twitter API. There are several Twitter API streams; we used the Academic Research Twitter API after submitting a project proposal to Twitter. This free-of-charge data collection method is no longer available due to recent changes in Twitter’s business model [27].

On November 30, 2022, we collected Twitter data using the R package “academictwitteR” [28]. We searched for tweets containing the terms “boysdocry” or “boysdocry2021” from all Twitter users worldwide. The search period included the 19 weeks of tweet activity while the campaign was active (during campaign) and the 19 weeks after the campaign ended (postcampaign). We excluded unrelated trends that involved our search term “boysdocry,” such as tweeting “boysdocry” to promote a song from the Eurovision Song Contest and tweets in languages other than English. Only variables of interest were stored for the analyses, including tweet ID, tweet text, date and time the tweet was created, type of tweet (original, quoted, and retweet), and reactions to the tweet (eg, number of likes, retweets, link clicks, and follows).

**Figure 2:**
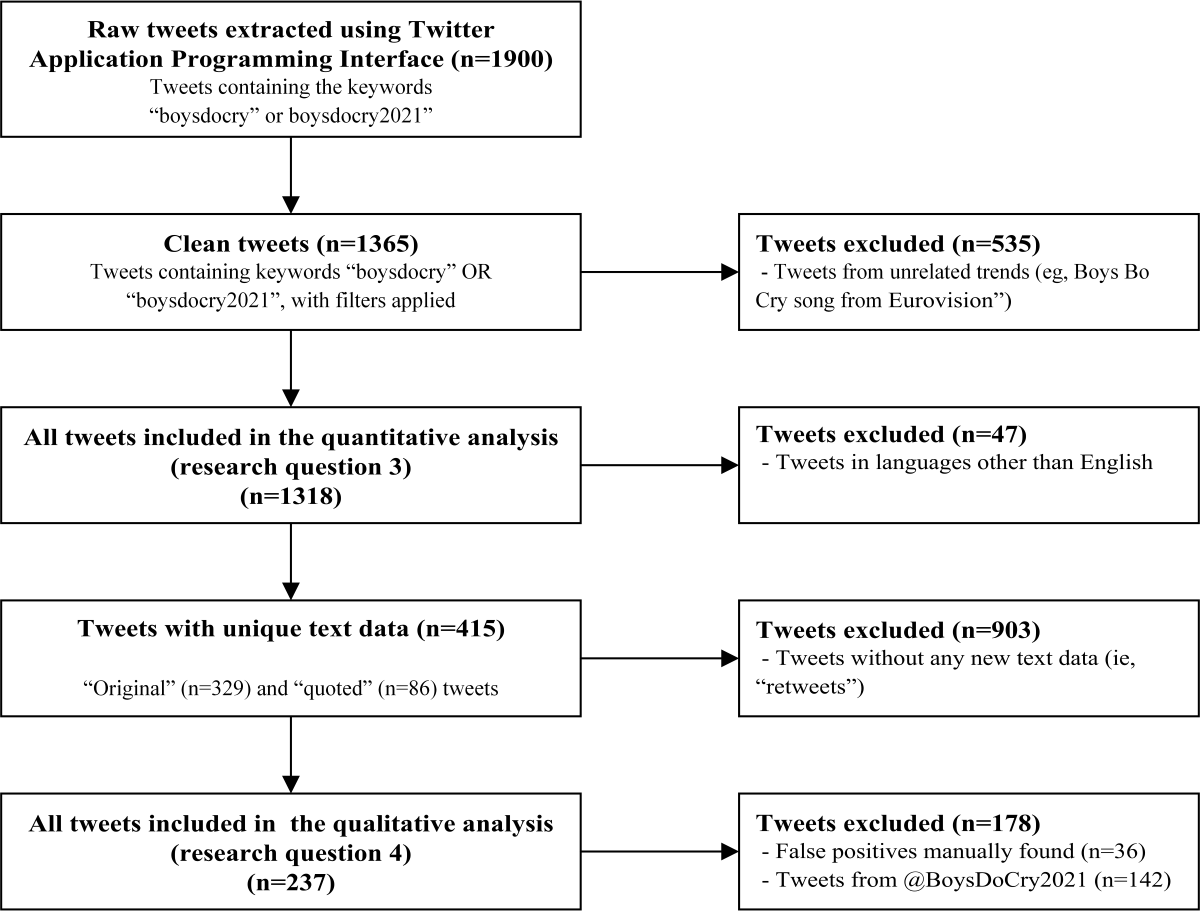
Inclusion and exclusion stages for the quantitative analysis (research question 3) and qualitative analysis (research question 4) for the Twitter Application Programming Interface data.

Figure 2 shows the flow diagram for the inclusion and exclusion stages (research questions 3 and 4) using Twitter API data. There were 1900 tweets containing the keywords “boysdocry” or “boysdocry2021” throughout the during-campaign period and postcampaign period. A total of 535 tweets were excluded as they were based on unrelated trends (eg, Boys Do Cry song from the Eurovision contest or from another singer). A further 47 tweets were excluded as they were in languages other than English, leaving 1318 tweets for the quantitative analysis in research question 3 using the Twitter API data. For the qualitative analysis (research question 4), we excluded 903 “retweets” as they did not include any new text data. However, we retained 86 “quotes,” as they are similar to retweets but with new text data added (ie, the user adds a comment in someone else’s tweet). During the data coding stage, we manually excluded 36 false positives and 142 tweets coming from the campaign hosts (@BoysDoCry2021). This left 237 tweets for the qualitative analysis.

### Data Analyses

We conducted quantitative analyses to answer research questions 1 to 3 and qualitative analysis to answer research question 4. For the analyses, we used R (version 4.2.2) [29] (package “tidyverse,” version 1.3.2 [30]) and Excel (Microsoft Corp).

For research questions 1 and 2, we used Twitter analytics data. For research question 1, we calculated the frequencies and averages for reach and engagement with the campaign during the campaign’s active period. For research question 2, we described tweet performance based on engagement with tweets posted by the campaign’s hosts, @BoysDoCry2021. First, we ordered all tweets posted by @BoysDoCry2021 based on the total number of times users reacted to those tweets. Second, we checked the 10 highest- and lowest-performing tweets and manually coded their content types. Tweet content type was based on their primary visual content, as we were interested in identifying what type of strategy (eg, attaching a visual component or having a purely text-based tweet) attracted more reactions, so tweets could be either a (1) video, (2) image, or (3) link. For instance, if a tweet involved an image or video and a link, we coded it as under image or video, so we checked all links to confirm if they involved any visual component. Videos were either embedded in the tweet or links to the campaign’s video (either through YouTube or through the campaign’s homepage). Tweets classified under “link” were therefore purely text-based content. We note that the 10 lowest-performing tweets included @BoysDoCry2021 replies or mentions to users without meaningful data (eg, “@username brilliant thread”), so we coded the next lowest-performing tweets with meaningful content in those instances.

For research question 3, we used the Twitter API data to calculate the number of campaign-related tweets per week and plotted their frequencies by campaign period (during-campaign vs postcampaign). Then we calculated the average number of tweets by campaign period. For research question 4, we used a subset of the Twitter API data to conduct the qualitative analysis. This data subset only included tweets in the English language. We excluded retweets as they did not have any new text data and had the potential to bias the results. We also excluded tweets coming from the campaign’s hosts, as our primary goal was to explore the audience discussions prompted by the campaign. We followed Braun and Clarke’s [31] methodology for thematic analysis, which allows identification of key themes by allocating codes to text. We conducted the thematic analysis of tweets in six stages: (1) SSR and AN independently read a random sample of 100 tweets and noted down initial ideas for codes representing major presenting themes; (2) we then discussed those initial codes and created a preliminary coding framework; (3) SSR and AN independently recoded the same sample using the established framework; (4) then we discussed any discrepancies and revised the coding framework; (5) we coded independently the remaining data using the revised framework and discussed further discrepancies until we reached consensus; and (6) we created a thematic map, which included the number of times tweets were coded under each theme.

### Ethics Approval

This study was approved by the Human Research Ethics Committee at the University of Melbourne (HREC No. 2022-25141-34361-3). All research was conducted in accordance with the National Health and Medical Research Council’s National Statement on Ethical Conduct in Human Research and the Declaration of Helsinki.

## Results

### Research Question 1: What Was the Reach of the Boys Do Cry Campaign and how did the Twitter Users Engage With it?

The 247 tweets posted by @BoysDoCry2021 were seen a total of 140,650 times by Twitter users during the campaign. Users engaged a total of 4477 times with those 247 tweets. Table 1 shows the total reach and engagement by campaign phase. Phase 1 had a considerably greater reach than phase 2; tweets from phase 1 were seen a total of 105,195 times, while tweets from phase 2 were seen a total of 35,455 times. Similarly, users engaged with @BoysDoCry2021 phase 1 tweets more frequently (a total of 3412 times) than tweets from phase 2 (a total of 1065 times).

**Table 1:** Summary statistics for reach and engagement with tweets posted by @BoysDoCry2021 during-campaign period by campaign phase.

		Phase 1^a^ tweets (n=116)	Phase 2^b^ tweets (n=131)	Total tweets (N=247)
**Reach^c^**				
	Users, n (%)	105,195 (74.8)	35,455 (25.2)	140,650 (100)
	Median (IQR)	196 (101-433.2)	133 (82.5-302.5)	159 (86-373)
	Minimum-maximum	8-27,529	9-2403	8-27,529
**Engagement^d^**				
	Users, n (%)	3412 (76.2)	1065 (23.8)	4477 (100)
	Median (IQR)	7 (3-13.5)	5 (2-11)	6 (2-12)
	Minimum-maximum	0-748	0-53	0-748

^a^Phase 1: “Watch the video” phase was from November 21 to December 31, 2021.

^b^Phase 2: “Take action” phase was from January 1 to March 31, 2022.

^c^Number of times Twitter users saw @BoysDoCry2021 tweets.

^d^Number of times Twitter users reacted to @BoysDoCry2021 tweets.

### Research Question 2: What Were the Highest- and Lowest-Performing Tweets and how did the Twitter Users Engage With Them?

A total of 9 out of 10 of the highest-performing tweets were from phase 1, while 7 out of 10 of the lowest-performing tweets were from phase 2. Table 2 shows the type of engagement with the 10 highest- and lowest-performing campaign tweets (in descending order of engagement) by content type.

**Table 2:** Types of engagement with the 10 highest and lowest performing tweets by content type.

		Highest-performing tweets			Lowest-performing tweets		
		Video^a^	Image^b^	Total	Link^c^	Image^b^	Total
Frequency, n (%)		5 (50)	5 (50)	10 (100)	7 (70)	3 (30)	10 (100)
**Sum of all engagements^d^, n**		1383	1093	2476	4	0	4
	Sum of detail expands^e^	395	180	575	0	0	0
	Sum of likes	237	216	453	3	0	3
	Sum of media engagements^f^	182	266	448	0	0	0
	Sum of URL clicks	224	136	360	1	0	1
	Sum of profile visits	158	182	340	0	0	0
	Sum of retweets	163	65	228	0	0	0
	Sum of hashtag clicks	15	46	61	0	0	0
	Sum of replies	5	2	7	0	0	0
	Sum of new followers^g^	3	0	3	0	0	0
	Sum of app opens^h^	1	0	1	0	0	0

^a^Embedded video or external link to video.

^b^Picture or screenshot.

^c^URL to external source that is not a video.

^d^Total number of times users have engaged with @BoysDoCry2021 tweets. “Engagement” refers to clicking anywhere on a tweet, including detail expands, likes, profile visits, retweets, hashtags, replies, new follows, app opens, and media engagements. See Twitter [26] for further details.

^e^Clicking or expanding a tweet's details.

^f^Engaging with a media-based tweet.

^g^New followers acquired based on a tweet.

^h^Clicking or opening external applications through a tweet.

All the 10 highest-performing tweets included some visual content, either a video (5/10, 50%) or an image (5/10, 50%). Likewise, the 10 highest-performing tweets involved sharing the campaign’s video or an image or screenshot promoting the campaign video. Among the 10 highest-performing tweets, the highest-performing tweet was the first tweet posted by @BoysDoCry2021, and it included the campaign’s core video embedded in the tweet.

Each day in Australia, an average of 7 men take their own lives. Together we can change the stats.

Introducing a new mental health campaign #BoysDoCry which aims to encourage men to get talking when they are struggling with their #MentalHealth.

https://t.co/9AVe8TYKaM

(with 748 engagements)

The second highest-performing tweet was a screenshot of the tweet posted by Robert Smith, the lead singer of The Cure, who wrote and performed the original song, shared the campaign’s video on YouTube and tagged the campaign account (ie, @BoysDoCry2021).

Huge 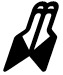 to @RobertSmith from @TheCure for sharing our adaptation of his song, and for supporting #mensmentalhealth!

Watch the music video now:

https://t.co/HOEkCBcfKU

#BoysDoCry2021 #RobertSmith #TheCure #GetTalking #MentalHealthMatters https://t.co/Hu2Y8xZkw7

(with 641 engagements)

The 10 lowest-performing tweets shared either a link (7/10, 70%) or an image (3/10, 30%). A total of 9 out of 10 of the 10 lowest-performing tweets involved sharing information related to masculinity, help-seeking resources, or broad resources such as articles and interviews. Examples of tweets posted by @BoysDoCry2021 with the lowest engagement are:

“When society creates an impossible standard of #masculinity to maintain, nobody wins.”

Interesting #newstudy published in Social Psychological and Personality Science @SPSPnews

<link>

#Relationships #Sociology

(with 1 engagement)

If you need support @LifelineAust have a 24/7 telephone counselling and crisis support line that’s free to call on 13 11 14. You can also visit our website for a list of organisations that can help on our website:

https://t.co/CuKsKcOUD5

(no engagements)

### Research Question 3: What was the Pattern of Twitter Activity for Campaign-Related Tweets Over the Course of the Campaign and in the Postcampaign Period?

In total, 1237 out of 1318 (93.9%) tweets were related to the Boys Do Cry campaign on Twitter in the during-campaign 19-week period (excluding campaign-generated tweets), while there were 81 out of 1318 (6.1%) campaign-related tweets in the postcampaign 19-week period. The summary statistics of weekly tweet counts by campaign period are shown in Table 3. The average number of weekly tweets during campaign was 24 (IQR 15.5-36), while the average number of weekly tweets for the postcampaign period was 2 (IQR 1.5-5). Multimedia Appendix 1 shows the campaign-related Twitter activity per week and by period (during-campaign and postcampaign). About half of the campaign-related tweets (n=608) were posted during week 1 of the campaign. The next highest campaign-related Twitter activities were during weeks 2 and 3, with over 130 tweets per week. During the campaign, three-quarters of all tweets were posted from weeks 1 to 4. Postcampaign, there were fewer than 10 campaign-related tweets in most weeks, except for weeks 25 and 26, which had 15 tweets each.

**Table 3:** Summary statistics of weekly tweet counts by campaign period^a^.

	During-campaign period	Postcampaign period
Frequency, n (%)	1237 (93.9)	81 (6.1)
Median (IQR)	24 (15.5-36)	2 (1.5-5)
Minimum-maximum	5-608	0-15

^a^Twitter Application Programming Interface data.

### Research Question 4: What Were the Key Themes of Discussion on Twitter Prompted by the Boys Do Cry Campaign?

Table 4 shows the 11 key themes that were constructed from the thematic analysis of the 237 unique campaign-related tweets. More than 90% of the qualitative coding fell under 5 themes.

The 237 campaign-related unique tweets were a subset of the Twitter Application Programming Interface data; it only included tweets in the English language and with new text data in (i.e., not retweets); see Figure 2 for further details.

**Table 4:** Thematic analysis of 237 campaign-related unique tweets.

Theme^a^	Description	Frequency, n (%)
Campaign support	Tweets showing support to the campaign, sharing the campaign video, or mentioning the campaign handle (@BoysDoCry2021).	235 (44)
Reference to original song/band/lead singer	Tweets mentioning the original song (Boys Don’t Cry), band (The Cure), or its lead singer (Robert Smith).	81 (15.2)
Reiterate the message	Tweets restating the campaign message, not just sharing the video or campaign link, or mentioning the campaign handle; also, tweets challenging traditional masculine ideas.	67 (12.5)
Emotional response	Tweets capturing emotional responses to the campaign or video, including using emotion-related emojis ( ** 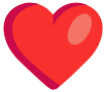 ** or ** 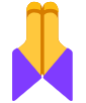 **) in the tweet and textual emotional reactions (eg, wow, beautiful).	65 (12.2)
Mental health-related hashtags	Tweets containing mental health-related hashtags (eg, #suicide, #suicideprevention, #mentalhealth, #mensmentalhealth).	45 (8.4)
Personal story	Tweets capturing someone sharing a personal story about themselves or someone close to them.	14 (2.6)
Support to others	Tweets showing support to other users.	10 (1.9)
Help-seeking resource	Tweets providing information or resource to help-seeking or encouraging the use of this resource.	7 (1.3)
Proud participant	Tweets about a proud participant involved in the campaign.	5 (0.9)
Negative to the campaign	Tweets criticizing or saying something negative about the campaign.	3 (0.6)
Seeking help	Someone asking for help.	2 (0.4)

^a^Tweets could be coded under more than one theme.

The most common theme was “campaign support” (235/534, 44.0%). This theme was about showing support for the Boys Do Cry campaign by sharing its video or the link to its website or mentioning the campaign handle (@BoysDoCry2021). Examples of tweets for campaign support are:

@BoysDoCry2021 We are more than happy to support such an amazing campaign 
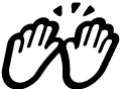
keep up the great work!

Such a powerful important message… to all my male #firefighter and #firstresponder colleagues and friends. This could save your life #SuicidePrevention #FRNSW https://t.co/ldHg9YQX4x

The second most frequent theme was “reference to original song/band/lead singer” (81/534, 15.2%). This theme included tweets that cited the original song, band or its lead singer. Examples of tweets coded under the reference to original song/band/lead singer theme are:

@RobertSmith @BoysDoCry2021 It's no surprise, but always a pleasure, to see you and The Cure supporting a strong cause 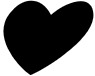
(but allow me to miss you and the guys so much)

@RobertSmith @username @BoysDoCry2021 Beautiful, I’ve always thought this is the emotion from where you wrote this song.

“Reiterate the message” was the third most common theme (67/534, 12.5%). This theme was related to tweets that incorporated core messages from the campaign around men reaching out for help when struggling and challenging traditional masculine ideas. Examples of tweets for reiterate the message are:

#boysdocry

when the going gets tough, get talking

Love this <link>

Sometimes old messages need replacing - this is one of those times. @BoysDoCry #MentalHealth https://t.co/WIJak25tSi

The fourth most frequent theme was “emotional response” (65/534, 12.2%). This theme involved tweets referencing emotional reactions in response to the campaign. Examples of tweets coded under this theme are:

Our men are broken. I know a lot over 40 that I worry about their mental health going forward. Hopefully hearing from other men to talk about their inner most feelings will help someone out there!

It feels awesome to have a big cry! Not a weakness but a strength https://t.co/fTNNqQwS7C

@RobertSmith @BoysDoCry2021 Heartwarming 
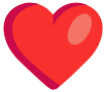

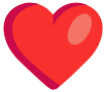

The fifth most common theme was “mental health-related hashtags” (45/534, 8.4%). The mental health-related hashtags theme included tweets with hashtags such as #suicide, #mentalhealth, and #mensmentalhealth. Examples of tweets coded under this theme are:

Absolutely spot on. Gents, it's OK not to be OK, and men DO cry. We lose far too many of you to suicide and we need to break the stigma NOW. #boysdocry #SuicidePrevention #MentalHealthMatters <link>

I’m listening

#BreakTheBias #Mentalhealth #boysdocry <link>

## Discussion

### Overview

This study is part of a larger evaluation of the Boys Do Cry campaign. In this study, we explored the campaign’s reach, engagement, and themes of discussion prompted by the Boys Do Cry campaign on Twitter. In assessing the content of conversations elicited by this campaign, we provide new insights regarding the critical elements for crafting future suicide prevention media campaigns targeting boys and men.

We found that the Boys Do Cry campaign reached a large number of Twitter users, prompting them to engage with its content. The most effective aspect for eliciting engagement was the addition of visual content in the campaign’s tweets. Evidence suggests that including visual content is an important strategy in public health communication because images have the potential to increase people’s attention to key health messages, especially when there is an emotional response to them [32]. Notably, the level of response to the campaign on Twitter was similar to the reach and engagement indicators observed in the campaign’s website analytics, suggesting that Twitter is a useful platform for hosting awareness and behavior change campaigns of this type.

Most public campaign-related Twitter activity occurred during the first week of the Boys Do Cry campaign, when an international celebrity—Robert Smith from The Cure—tweeted about the campaign. However, it is also likely that many campaign followers were more interested in the campaign when it was new. This may be a characteristic of this type of health promotion campaign; for example, another study monitoring suicide and depression discussions on Twitter over time (176 million tweets) found that Twitter activity faded quite rapidly following expected events, such as mental health campaigns, compared to unexpected events, such as a celebrity death by suicide [33].

This study also examined the key discussion themes prompted by the Boys Do Cry campaign. Key among these themes was that people supported the campaign and reinforced its messages. This finding may reflect a desire to challenge masculine norms, such as the notion that boys and men should not cry and should keep their emotions in check, even in difficult times. Evidence suggests that the masculine norm of self-reliance is associated with suicidal thinking in men [15] and thus may be a target for interventions to reduce suicidal thinking and acts. Another major topic of discussion prompted by the campaign was related to the original song by The Cure, Boys Don’t Cry. It is possible that a well-known song by a famous band with a prominent lead singer may have assisted in promoting the campaign’s message. There are suggestions from elsewhere that celebrities may play an important role in the delivery of messages in public health media campaigns (Brown and De Matviuk [34] and Moskowitz et al [35]).

Our findings are in part similar to those from our Twitter evaluation of another suicide prevention media campaign focused on challenging traditional masculinity, called “Man Up” [18]. We note that the core component of the Man Up campaign was a 3-part documentary that included nearly 3 hours of content, while the Boys Do Cry campaign had a 4-minute music video at its center. Despite this difference, we found that the Boys Do Cry campaign did promote discussions on the importance of shifting old views related to traditional masculine norms in the same way reported for Man Up. Examples of tweets with this sentiment related to the Boys Do Cry campaign included: “Sometimes old messages need replacing - this is one of those times”; “when the going gets tough, get talking.” Perhaps because of its longer format and opportunity to explore more issues, the Man Up campaign elicited a broader range of themes and additional discussion around masculine norms (eg, what it means to be a man, father, or raise boys [18]) compared to the Boys Do Cry campaign.

### Limitations and Strengths

This study had some limitations. Our search query for the Twitter API only searched for the string “boysdocry” (ie, “boysdocry” or “boysdocry2021”), so it is possible that we did not collect tweets that used the term “boys do cry” with spaces. However, our pilot searches revealed that the inclusion of spaces in the query would return more false positives than we could feasibly manage manually, so we did not include spaced terms. Another limitation is the potential bias in this study sample based on the demographics of Twitter users, as there are documented differences on social media preferences by age group [36]. This study also had some strengths. This is one of the few studies to evaluate a suicide prevention media campaign targeted at men using Twitter data [18,20]. Finally, we used a rigorous method of data collection—the Twitter API—to conduct this study and understand the key topics of discussion prompted by the Boys Do Cry campaign.

### Conclusions

Social media platforms offer another avenue through which to engage with boys and men on the topic of masculinity and suicide. This study demonstrates that a brief media campaign such as Boys Do Cry can achieve good reach and engagement and can prompt discussions on Twitter around the issue, which may lead to greater awareness about the importance of seeking help and providing support. However, this study suggests that longer, more intensive campaigns may be needed in order to amplify and sustain these results.

## Appendix

Multimedia Appendix 1 Number of tweets per week during campaign and postcampaign periods.

## Abbreviations

API: application programming interface

## Data Availability

The data collected and analyzed during this study are available from the corresponding author on reasonable request.
