# Supplementary figures and images for: An Evaluation of the Boys Do Cry Suicide Prevention Media Campaign on Twitter: Mixed Methods Approach
Source: JMIR Form Res. 2023 Sep 7;7:e49325. doi: 10.2196/49325 (PMC10514762; doi:10.2196/49325)

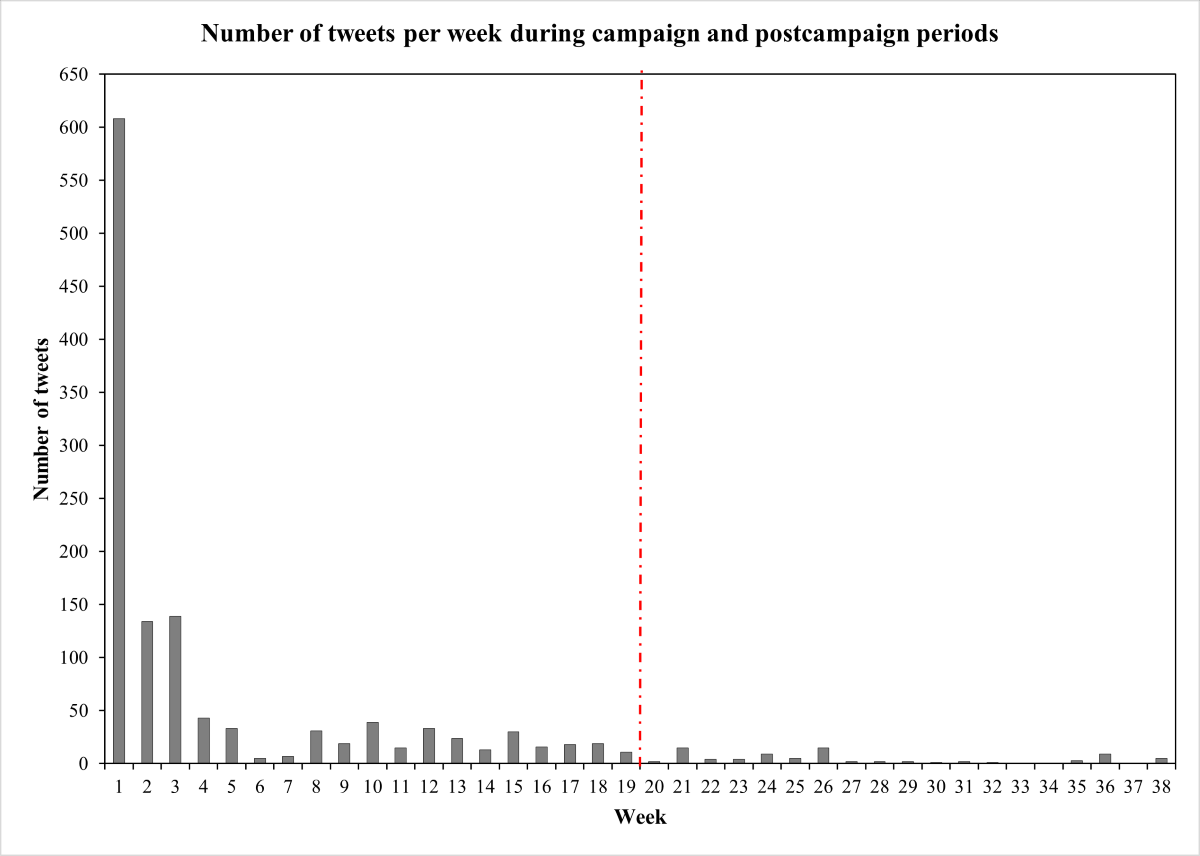

Supplement: Multimedia Appendix 1 [file formative_v7i1e49325_app1.png]
